# Twelve tips for the transition from training to first faculty position

**DOI:** 10.12688/mep.20391.2

**Published:** 2025-05-12

**Authors:** Beatrice Preti, Michael Sanatani

**Affiliations:** 1 Division of Medical Oncology, Western University, London, Ontario, Canada; 2 Department of Haematology & Medical Oncology, Emory University, Atlanta, Georgia, USA

**Keywords:** professional identity formation, continuing professional development, transition to practice, resident, fellow, consultant, attending

## Abstract

The transition from residency or fellowship to autonomous, independent consultant can be daunting, to say the least! New consultants may face a number of challenges and decision points previously unencountered in their careers. In this article, we present twelve tips for trainees transitioning to their first consultant position (with an emphasis on those in academic or hybrid positions) to help with a smooth, successful process.

## Introduction

Medical education consists of a stepwise progression between roles: from layperson to medical student, from student to resident, and, ultimately from resident to autonomous, independent consultant (or attending staff). Each of these transitions brings with it unique challenges for the developing physician as a person, a process well-rooted in previous professional identity formation theory ^1^. The professional identity development process, for example as described by Cruess ^2^, is however intertwined with practical challenges and aspects of independent practice, which are the focus of this article. While formal supports in training programmes may assist with the first two transitions (into medical school, and then into residency), the progression from postgraduate trainee to autonomous consultant can also be a difficult process for a physician to navigate ^3, 4^. However, unlike previous transitions, and depending on the new consultant’s position and work milieu, formal supports for this transition can be minimal to absent, leaving the new consultant to walk this path in more relative solitude than they are accustomed to.

And yet, the transition to consultancy does come with its own steep learning curve. New attendings may grapple with leadership skills, decision-making (including the weight of clinical decisions they now bear full responsibility for), balancing teaching and patient care, balancing service obligations, balancing budgets and repaying loans, and more ^5^. Proper mentorship and attention to weak points can assist with a successful transition, allowing the physician to thrive ^6^. It is common for academic centres, at least, to have formal mentorship programmes for faculty members; however, these can vary in usefulness for a new consultant, especially one in a new centre where the consultant’s history and reputation are not known, and advice/counselling may be generic, untargeted, or clash between the goals/personalities/communication styles of mentor and mentee ^7^. Effective mentorship takes time to develop and grow ^8^. Consequently, using formal, assigned mentorship may be initially insufficient as a lone resource for the new consultant. However, with the proper mindset in both mentor and mentee, the transition to consultancy can be a period of great personal growth and learning ^9^, paving the way for future successes. Reciprocally, a great deal of a physician’s career trajectory hinges on the results of the first few years, and failure to achieve key skills and balance may negatively impact the physician’s career as a whole.

Inspired by Kegan’s model of development, we present twelve practical tips to assist with the transition from trainee to consultant, based on the published literature, including cited articles and books ^10^, as well as our own experiences as an experienced consultant and mentor (MS) and a new consultant six months post-graduation (BP). While these tips are written directly to the new faculty member making the transition from training, these tips can also be used by mentors and leaders guiding the fledgling consultant.

## Tip 1: Ask for help when needed

Throughout training, we’ve all heard a gentle reminder (or, perhaps, not so gentle!) to ask for help when needed. This piece of advice certainly has its place in the transition to consultancy as well. The specifics of the “help” may vary; while your clinical knowledge will have progressed from the first few days as a first-year resident, you can face equally-daunting challenges as you navigate a new department, electronic medical record (EMR), hospital site (and obtaining necessary credentialing and licensure), or even clinic space and associated staff. Simultaneously, it’s important to identify senior colleagues and peers you feel able to ask for advice about cases or challenging patient situations, especially when working in a new department where the workflow or preferred diagnostic/treatment strategies may differ. Collaboration between physician colleagues has been shown, logically, to improve patient outcomes and satisfaction scores ^11^, although especially in an academically competitive environment it is important to ensure that sharing and attribution of ideas between peers is properly acknowledged by all parties, should they have a connection to ongoing academic or clinical work.

The importance of having strong, individualised mentorship for the transition to consultancy cannot be understated ^12^. This will likely consist of a variety of mentors who provide different supports (including clinical expertise, administrative expertise, job/career advice, even peer mentors to debrief with), and seeking out such individuals is strongly encouraged. External mentors outside one’s department or even institution may also provide valuable perspectives, and while they may not be as familiar with the specific challenges in the local context, they would provide a safe space free of potential conflicts of interest. New consultants should be encouraged to cultivate such relationships (perhaps through a professional organisation, networking at conferences, or through mutual acquaintances/professional interests).

## Tip 2: Seek out opportunities that interest you/are in line with your goals

As a new consultant, you will find potential opportunities and requests abound. These can range from research projects and ideas, committee involvement, teaching commitments, administrative positions, quality improvement initiatives, and, of course, (sometimes, seemingly never-ending) service and patient care obligations. The sheer number of possibilities can be overwhelming, and the undiscerning young consultant runs the risk of overcommitting – and, consequently, rendering themselves unable to complete *any* task to satisfaction ^13^.

Even though all physicians face necessary tasks they might find mundane or undesirable, it’s a good idea to write down areas of personal interest where the you are hoping to grow your career. Ask questions like:

- *What brings me so much joy that I can lose track of time doing it?* This may suggest an area or areas within your work to explore further.
- *What brings me a sense of professional (or personal!) fulfilment and satisfaction?* These may be areas where adding responsibilities may contribute positively to a sense of wellbeing, rather than being seen as just another task to accomplish.
- *Where do I see myself in 5 years? 10 years? 20 years? At retirement? Is there any place I DON’T want to end up?* Creating a (very!) tentative roadmap can help provide direction and planning for the future.

Knowing what you hope to achieve over time can serve as a guide during the early days of consultancy, allowing options to be sorted through in order to figure out which ones will be most helpful down the road. This is also a good time to network and try to uncover opportunities that perhaps weren’t available or recognised during training. Mentors can also be helpful in pointing out promising networking opportunities or making introductions.

At the same time, it’s important to remember that every position and role comes with responsibilities and expectations which might not be fully in line with your personal/professional interests. Being a team player, while pursuing (and protecting time for) one’s own interests and goals, is a balance every consultant must learn to strike.

## Tip 3: Practise effective boundary setting

In order to protect again burnout and related conditions, it is crucial for every young consultant to set themselves along a sustainable path, one that ideally could be pursued for decades. If you can’t imagine doing your current job for the next thirty years (and there isn’t an immediate crunch, such as a leave being covered, or a period before a new hire), some change is needed.

Setting boundaries, both in and out of work, is a necessary component of any sustainable medical career ^11^. This may be a particularly challenging mind-shift for a former trainee who is used to “auditioning” for consultants, perhaps for evaluations, reference letters, or career advancement opportunities. However, such levels of stress and impression management are not sustainable long-term, and carving out time and space for the individual is imperative. For most of us, boundaries with time outside of work (and, for academic work, outside of patient care) are crucial. This may include choosing not to have work email on one’s cell phone, prioritising family commitments over social work events, or not checking EMR notifications during vacation time. Some young consultants may choose to join a working group, where a group of academics gather either in-person or online for time dedicated to academic work. Others may choose to manage their own schedules, grouping meetings to ensure time between for administrative or academic work. Also consider institutional and job policy regarding protected time and pager/patient coverage when searching for positions – and don’t be afraid to advocate if promised things are not delivered.

Inherently, boundary-setting may be in conflict with following Tip #2, which encourages pursuing new opportunities with enthusiasm. While there is no simple recipe for striking the correct balance, it is exceedingly important to keep this balance in mind. In most contexts, it is patently impossible for a consultant (new or old) to perfectly satisfy everything that would, in theory, be required of them, and still sleep at night. The consultant may have to grow a “thick skin” and leave some things intentionally incomplete or delayed so that higher-priority pursuits can go on, something which represents a paradigm shift from training. While navigating this situation goes beyond the scope of this article, the reader is referred to the copious existing literature on reconciling academic work pursuits and personal sustainable workload. In our experience, new consultants tend to overachieve, so the idea of “lifelines” instead of “deadlines” might hold merit – in other words, set dates at which specific projects come to life (begin), and do not start working on it beforehand.

## Tip 4: Remember to build relationships with administrative assistants, nurses, allied health, and clerical staff

Particularly when starting at a new centre, the newly-minted consultant may be tempted to focus on building collegial relationships with other physicians, both in and out of their department, as they try to develop a solid reputation as a clinical provider and in other areas of professional expertise, such as a research track. However, it’s important to remember the key players who will assist with day-to-day tasks and, especially, patient care. We cannot underscore enough the importance of taking time to get to know the non-physician team members. Ask questions that show you value their opinions and contributions to the team. Ask for feedback from the team regularly as well, especially when working with seasoned individuals who have worked with other physicians or in other healthcare settings before. Don’t be afraid to say if something works or doesn’t work for you, but also remember those around you have experience, opinions, and ways they may prefer to do (or have you do) tasks as well. Conflict is inevitable and may often be based on a genuine difference in belief of what is in a patients’ best interest, but also on a reluctance to try a new approach if what has always been done has, in their view, also always worked. In addressing tensions, do not hesitate to speak to the physician you report to (chief, section head), following chief, the established approach for raising concerns ^14^.

## Tip 5: Don’t lose your nerve

The start to consultancy can be rocky. You *will* make mistakes. You will run behind schedule, or forget certain tasks, or miss deadlines. You won’t know everything, and you’ll forget things you thought you knew. Patients may be irritable, preferring to see more experienced clinicians, or clinicians of certain demographic backgrounds. You will likely get patient complaints, including about things out of your control, such as wait times or treatment side effects. Other healthcare workers may also be irritable, and show low levels of patience for mistakes or inefficiency due to your junior status.

Throughout all of this, it's important to remember to be kind to yourself. You are going through a challenging period of time – as are many of the people around you. Ensure you make time at the end of the day to debrief with your support system and disconnect from the daily grind. Make time to eat, sleep, spend time with loved ones, and rekindle old hobbies, perhaps put on hold during residency. A degree of resilience is required for any healthcare provider, and the new consultant is no exception. Having a “game plan” in advance of the transition (i.e. setting up regular meetings with friends, joining a sports league, planning a family holiday six months into the job) can be helpful. In addition, semi-formal meetings in the work context to address situations when things went “sideways” can be helpful to normalize your experience ^15^! One of the new consultants at our institution initiated a bi-monthly “morbidity and mortality” rounds program to discuss difficult outcomes when they started, and it has become a very popular and effective discussion platform even for the senior members of the department.

## Tip 6: Manage your time

Managing your time as a consultant is crucial to success. Days can fill up with numerous “little tasks” (emails, pages, phone calls, orders to sign, notes to cosign) which can drain productivity and energy – especially if left to accumulate until the end of a busy workday, eating into valuable family or home time. Using “whitespace” ^16^, or the downtime between major tasks, to complete these little tasks can boost efficiency. Consider, for example, sending a quick reply to an email while awaiting an elevator, or reviewing the EMR in-basket while waiting for the next clinic patient to arrive. Not only can this make you be seen as more responsive and responsible, but it can also help with efficiency.

At the same time, many tasks (both clinical and academic) require uninterrupted periods of concentration, where even a knock on the door, phone call, or email can cause a setback. Ensure appropriate planning (whenever possible) to protect blocks of time for this type of work. For example, consider asking clinical staff to batch non-urgent requests into designated times – for example, between patient visits – instead of interrupting notes or orders, possibly leading to distraction or even errors. Consider scheduling morning meetings back-to-back to allow for several free hours for academic writing in the afternoon, or putting a sign on the door for urgent interruptions only while making patient calls, along with a notepad for callbacks, etc. The possibilities are endless, although it may take some tries (and a lot of communication!) to figure out a strategy which works best for you. Some common strategies include both environment- or work-based strategies ^17^, as well as more personalised strategies which might be learnt through mentors, coaches, or workshops ^18^.

## Tip 7: Physicians are lifelong learners – in more ways than one!

During medical training, learners are primarily taught to focus their studies around patient care (i.e. the ubiquitous “read around cases”). While consultants do need to read and study to keep abreast of their medical knowledge, their expanded role will also likely require study in other domains, such as teaching and research skills, depending on the consultant’s area of focus. Naturally, no single consultant is able to develop mastery in all areas they may encounter through their day-to-day work; however, a basic degree of competence may need to be sought out, particularly if skills were not formally taught during residency. On the flip side, however, any one consultant can only be an “expert” in a few areas, and seeking out self-development opportunities and courses in line with one’s interests can help develop mastery – a learning process which will need to continue throughout the consultant’s career.

## Tip 8: You alone are responsible for your actions

Consultants are independent, autonomous healthcare providers. While this can be an exciting time, the weight of your actions is heavier now than previously during residency, when working under the supervision of a more senior physician, and mistakes and missteps can have a higher cost ^19^.

While soliciting the opinions and advice of others is encouraged, it must be remembered that you alone are responsible for your own actions. Bear in mind that any case you see, including any order, email, or note you write, may end up having to be defended in front of a judge – and act accordingly. Likewise, both in your personal and professional lives, consider that your words can be used as medical advice; how would your social media post or email sound in a headline (“Doctor Says X”)? What is your institution’s attitude or regulations regarding public or social media content? In general, it is advisable to separate your clinical practice from social media, especially as it concerns potentially identifiable scenarios. As trainees, the shield of a consultant’s ultimate responsibility is removed – you are the consultant now, which comes with its own weight. Keep this in mind during documentation of any clinical or professional encounter.

## Tip 9: Be open to new possibilities

Prior research has shown that newly-minted consultants find having an open mind, and being flexible and adaptive in the workplace, aided their transition from residency to consultancy ^20^. The specifics of an “open mind” may vary from simply figuring out a more efficient way for clinic flow, accepting feedback and reconsidering practises learnt in residency, or perhaps trying a new role or research project to see how it might align with personal goals. The roadmap to the future is always in flux, and testing new possibilities can lead to new roads, pit stops, and destinations previously not considered.

## Tip 10: Take care of your own health

Medicine is draining. With new responsibilities and additional stressors, the new consultant may find themselves more drained at the end of the day than they are previously accustomed to. It is impossible to complete every task you will face on a daily basis – even within the work domain alone. It is also necessary to conserve energy for out-of-work responsibilities: family life, household chores, socialising, exercise, and routine health needs. Consultancy is a marathon, not a sprint, and setting limits while prioritising what matters to you can help make for a more sustainable career.

Furthermore, it can be easy to become distracted and focussed on work, to the detriment of your own physical/mental health. When planning your schedule, ensure you carve out time for food/hydration (including when working), exercise, hobbies, and relaxation. Also ensure you make time to focus on your own medical needs, including establishing with a family physician (and seeing them as needed!), as well as seeking out counselling or therapy sessions, if needed.

## Tip 11: Be assertive. Don’t say “yes” to things that don’t work for you

Without the protection of a residency body, union, programme administrator, chief residents, programme director, and more, it can be easy for the new consultant to feel coerced into taking on extra tasks or roles, especially when working in certain spaces where there may be an expectation for more junior members to take on more work. Likewise, when working with a new clinical team, there may be requests for altered work flows (for example) which the new consultant has difficulty incorporating into their practice. Often, physicians may feel obligated to accommodate requests, especially under the guise of forming or maintaining good relationships, or being seen as likeable or a hard worker. In determining whether something “works” for you, while also having some open-mindedness, it may be helpful to refer back to some of the self-reflective questions in Tip #2 and Tip #3 – does this new activity align with the direction you envision for your career? Or does it distract you from patient care or professional development?

Communication and compromise in these situations are key. Remembering that healthcare is provided by a team, working with the team to find solutions that are acceptable to everyone is the best way to ensure everyone is satisfied with the result. Likewise, work and tasks should be appropriate to one’s role. Young consultants struggling in this area may find benefit, again, from coaching or coursework to build skills in assertiveness.

## Tip 12: Find your tribe

Throughout this article, numerous references have been made to a support network, mentors, peers, and trusted individuals, both in and out of medicine. No longer under the wing of a postgraduate training programme, you may find your support system has changed or decreased in size.

This is normal and natural – relationships evolve over time, and people may grow apart. However, this does not make having a support system any less important. Now is the time to spread your own wings and find your people. Network at conferences in areas of interest. Sign up for mentorship programmes. Join online communities, if they exist within your field – or create your own! Explore life outside medicine to find like-minded individuals you can connect with about other areas of interest.

## Conclusions

We have presented twelve tips for new consultants making the transition from residency to autonomous practice. Key tips include creating and maintaining a support network, maintaining boundaries, and exploring options with a flexible path in mind. We hope these tips are useful for the new generation of fresh consultants, and would be delighted to hear how our readership experiences implementing these tips!

## Data Availability

No data are associated with this article.

## References

[1] Lewin LO McManamon A Stein MTO : Minding the form that transforms: using kegan's model of adult development to understand personal and professional identity formation in medicine. *Acad Med.* 2019;94(9):1299–1304. 10.1097/ACM.0000000000002741 31460919

[2] Cruess RL Cruess SR Boudreau JD : Reframing medical education to support professional identity formation. *Acad Med.* 2014;89(11):1446–51. 10.1097/ACM.0000000000000427 25054423

[3] Allen BR : Transition to practice: from resident to faculty at the same institution. *J Grad Med Educ.* 2014;6(4):799–800. 10.4300/JGME-D-14-00266.1 26140141 PMC4477588

[4] van Delft KWM Nightingale G : The transition from resident to consultant. *Int Urogynecol J.* 2019;30(8):1219–1220. 10.1007/s00192-019-04000-0 31197427

[5] Roten C Baumgartner C Mosimann S : Challenges in the transition from resident to attending physician in general internal medicine: a multicenter qualitative study. *BMC Med Educ.* 2022;22(1): 336. 10.1186/s12909-022-03400-z 35501754 PMC9063076

[6] Okereke I : Commentary: the transition from resident to attending is a marathon, not a sprint. *J Thorac Cardiovasc Surg.* 2020;159(3):1161–1162. 10.1016/j.jtcvs.2019.09.096 31668535

[7] Straus SE Johnson MO Marquez C : Characteristics of successful and failed mentoring relationships: a qualitative study across two academic health centers. *Acad Med.* 2013;88(1):82–9. 10.1097/ACM.0b013e31827647a0 23165266 PMC3665769

[8] Hill SEM Ward WL Seay A : The nature and evolution of the mentoring relationship in academic health centers. *J Clin Psychol Med Settings.* 2022;29(3):557–569. 10.1007/s10880-022-09893-6 35761033 PMC9243938

[9] Westerman M Teunissen PW van der Vleuten CP : Understanding the transition from resident to attending physician: a transdisciplinary, qualitative study. *Acad Med.* 2010;85(12):1914–9. 10.1097/ACM.0b013e3181fa2913 20978429

[10] Roberts LW : The academic medicine handbook.Springer,2013. 10.1007/978-1-4614-5693-3

[11] Braam A Buljac-Samardzic M Hilders CGJM : Collaboration between physicians from different medical specialties in hospital settings: a systematic review. *J Multidiscip Healthc.* 2022;15:2277–2300. 10.2147/JMDH.S376927 36237842 PMC9552793

[12] Harrison R McClean S Lawton R : Mentorship for newly appointed physicians: a strategy for enhancing patient safety? *J Patient Saf.* 2014;10(3):159–67. 10.1097/PTS.0b013e31829e4b7e 24583961

[13] Patel RS Bachu R Adikey A : Factors related to physician burnout and its consequences: a review. *Behav Sci (Basel).* 2018;8(11):98. 10.3390/bs8110098 30366419 PMC6262585

[14] Bochatay N Bajwa NM Cullati S : A multilevel analysis of professional conflicts in health care teams: insight for future training. *Acad Med.* 2017;92(11):S84–S92. 10.1097/ACM.0000000000001912 29065028

[15] MacMillan TE Rawal S Cram P : A journal club for peer mentorship: helping to navigate the transition to independent practice. *Perspect Med Educ.* 2016;5(5):312–5. 10.1007/s40037-016-0292-2 27631332 PMC5035278

[16] Arrow K Resnik P Michel H : Evaluating the use of online self-report questionnaires as clinically valid mental health monitoring tools in the clinical whitespace. *Psychiatr Q.* 2023;94(2):221–231. 10.1007/s11126-023-10022-1 37145257 PMC10160731

[17] Kobza J Syrkiewicz-Świtała M : Time Based Management in health care system: the chosen aspects. *Med Pr.* 2014;65(4):555–66. 10.13075/mp.5893.00050 25643493

[18] Pitre C Pettit K Ladd L : Physician time management. *MedEdPORTAL.* 2018;14: 10681. 10.15766/mep_2374-8265.10681 30800881 PMC6342364

[19] Gurley KL Grossman SA Janes M : Comparison of Emergency Medicine malpractice cases involving residents to nonresident cases. *Acad Emerg Med.* 2018;25(9):980–986. 10.1111/acem.13430 29665190

[20] Kahn JM DiazGranados D Fields EC : Transitioning roles from residency to attending physician in radiation oncology. *J Cancer Educ.* 2022;37(4):1179–1185. 10.1007/s13187-020-01936-6 33415650 PMC8263787

