# Supplementary material for: Twelve tips for the transition from training to first faculty position
Source: MedEdPublish (2016). 2025 May 12;14:42. Originally published 2024 Jun 3. [Version 2] doi: 10.12688/mep.20391.2 (PMC12144489; doi:10.12688/mep.20391.2)
Supplement: Peer review history

# Reviewer report: Reviewer response for version 2

**DOI:** 10.21956/mep.22076.r41774

**Published:** 2025-06-06

**Authors:** Stella Yiu

**Affiliations:** 1 Department of Emergency Medicine, The Ottawa Hospital Foundation, Ottawa, Ontario, Canada

The manuscript is okay to be indexed.

If evidence from practice is presented, are all the underlying source data available to ensure full reproducibility?

Yes

Is the topic of the practical tips discussed accurately in the context of the current literature

Partly

Are all factual statements correct and adequately supported by citations?

Yes

Are the conclusions drawn balanced and justified on the basis of the presented arguments?

Yes

Are arguments sufficiently supported by evidence from the published literature and/or the authors’ practice?

Partly

Reviewer Expertise:

NA

I confirm that I have read this submission and believe that I have an appropriate level of expertise to confirm that it is of an acceptable scientific standard.

# Reviewer report: Reviewer response for version 1

**DOI:** 10.21956/mep.21821.r37971

**Published:** 2024-08-13

**Authors:** Stella Yiu

**Affiliations:** 1 Department of Emergency Medicine, The Ottawa Hospital Foundation, Ottawa, Ontario, Canada

Thank you for taking the time to write this based on your experience. I enjoyed reading it. I think it will be a useful report for those entering into newly independent practice.

 Overall: Like the tips and the recommendations. The article might benefit from more practical resources, and more references to strengthen arguments.

**Abstract:**

 - great topic with a clear emphasis on the objective

 - 'consultant' term might not be universal. Perhaps explaining it further would be helpful (is that equivalent to attending?)

**Introduction**

 - First paragraph: Good introduction to professional identity formation. Is the emphasis for the article on PIF or the transition, or both? Perhaps explaining the focus would make it clearer for the reader. If the emphasis is on PIF, then perhaps adding references from the body of work by Cruess might strengthen the argument

 - Second paragraph: Agree with extra non-academic tasks for independent practitioners, and literature discussing mentorship as a viable option to ease this transition. Would appreciate more clarity and explanation for this point: does that point at mentorship as great but insufficient? Is this point leading to 'what else should the individual do besides formal mentorship'? (Might be helpful to include the 2018 Yardley article.)

 - Third paragraph: Is this professional development theory mentioned Kegan's model? or is it the professional identity formation theory? 

 I appreciate its audience being both established clinicians (potential mentors) and also newly independent physicians

 Tip 1: Agree mentorship is a very helpful step. Perhaps some discussion of pros and cons of having internal/external mentors and near peer/senior (if space allowed)? 

 Tip 2: Useful questions to consider. 

 Tip 3: I imagine new (and many others) physicians having trouble balancing Tip 2 and 3. How would one navigate that? Could you explain what you mean by radical?  

 Tip 4: Great tip. Change MD to physicians? What happens if there is conflict? How should they raise their objections as a new member? (I have added a conflict paper - perhaps it could be of use?) Who should they talk to?

 Tip 5: Agree with this point. Also that new physicians often think they are the only ones making mistakes, so having discussion with peers to normalize their experience might help.  The MacMillan article might be useful.

 Tip 6: great point. Really like the second paragraph of this tip. Adding links to resources on time and project  management would be even more helpful

 Tip 7: Agree wholeheartedly on this point

 Tip 8: Perhaps separating social media from clinical practice would emphasize it more as it is an important point. 

 Tip 9, 10, 11: great points - how do new physicians judge things that 'work'? What tools can they use?

If evidence from practice is presented, are all the underlying source data available to ensure full reproducibility?

Yes

Is the topic of the practical tips discussed accurately in the context of the current literature

Partly

Are all factual statements correct and adequately supported by citations?

Yes

Are the conclusions drawn balanced and justified on the basis of the presented arguments?

Yes

Are arguments sufficiently supported by evidence from the published literature and/or the authors’ practice?

Partly

Reviewer Expertise:

Tacit knowledge in newly independent physicians, organizational culture.

I confirm that I have read this submission and believe that I have an appropriate level of expertise to confirm that it is of an acceptable scientific standard, however I have significant reservations, as outlined above.

## References

[1] : The do's, don't and don't knows of supporting transition to more independent practice. *Perspect Med Educ* .2018;7(1) : 10.1007/s40037-018-0403-3 8-22 10.1007/s40037-018-0403-3 29383578 PMC5807269

[2] : Supporting the development of a professional identity: General principles. *Med Teach* .2019;41(6) : 10.1080/0142159X.2018.1536260 641-649 10.1080/0142159X.2018.1536260 30739517

[3] : A Multilevel Analysis of Professional Conflicts in Health Care Teams: Insight for Future Training. *Acad Med* .2017;92(11S Association of American Medical Colleges Learn Serve Lead: Proceedings of the 56th Annual Research in Medical Education Sessions) : 10.1097/ACM.0000000000001912 S84-S92 10.1097/ACM.0000000000001912 29065028

[4] : A journal club for peer mentorship: helping to navigate the transition to independent practice. *Perspect Med Educ* .2016;5(5) : 10.1007/s40037-016-0292-2 312-5 10.1007/s40037-016-0292-2 27631332 PMC5035278

# Reviewer report: Reviewer response for version 1

**DOI:** 10.21956/mep.21821.r37805

**Published:** 2024-08-13

**Authors:** Colleen Gillespie

**Affiliations:** 1 NYU Langone Health, New York, NY, USA

This was a well written, clearly delineated, and very helpful "how to" guide for new faculty in the "12 tips" format.

 A few minor suggestions:

 Introduction

 I think it is worth acknowledging two additional challenges of transitioning to the faculty role:

 1) Often learning a new healthcare system, setting etc (this then helps serve as the set up for Tip 4 - Build relationships with team) and 2) building new support and information/advice networks

 Tip 1:  Ask for Help

 The second paragraph doesn't quite fit into "ask for help" -  I would recommend expanding the tip and the initial set up to continue to emphasize "asking for help" but then explain more directly that in the faculty role the next level of asking for help is building support networks that provide ready access to help (this then sets up the having great mentorship).  I wonder if a line or two about how to be a good mentee could be useful here (or referencing an appropriate article about the mentee side of mentorship).

 I felt like Tip 6 and Tip 5 could be swapped -- for me, having Manage your time before Don't lose your nerve made more sense to me.  Similarly, maybe Tip 7: Physicians are lifelong learners could be moved to be closer to Tip 2-Ask for help or Tip 5-Don't lose your nerve.  

 I recommend expanding Tip 10 to read: Taking care of your self and your health  -- just to broaden it a bit.

 And in the abstract, I'd be careful about arguing that these tips could help with a "smooth" process -- that's probably a bit much to ask!  Maybe "to help these new faculty successfully take on this challenging and rewarding role" or something like that.

If evidence from practice is presented, are all the underlying source data available to ensure full reproducibility?

Yes

Is the topic of the practical tips discussed accurately in the context of the current literature

Yes

Are all factual statements correct and adequately supported by citations?

Yes

Are the conclusions drawn balanced and justified on the basis of the presented arguments?

Yes

Are arguments sufficiently supported by evidence from the published literature and/or the authors’ practice?

Yes

Reviewer Expertise:

I am a social scientist by training - not an MD or clinician -- so can only comment on the usefulness of this article based on my observations of my many junior faculty colleagues.

I confirm that I have read this submission and believe that I have an appropriate level of expertise to confirm that it is of an acceptable scientific standard.
